# Association of high disease activity and serum IL-6 levels with the incidence of inflammatory major organ events in Behçet disease: a prospective registry study

**DOI:** 10.3389/fimmu.2024.1354969

**Published:** 2024-04-15

**Authors:** Lisa Hirahara, Yohei Kirino, Yutaro Soejima, Yuki Iizuka, Ryusuke Yoshimi, Yuichiro Fujieda, Tatsuya Atsumi, Toshihiro Tono, Daisuke Kobayashi, Akira Meguro, Masaki Takeuchi, Kentaro Sakamaki, Mitsuhiro Takeno, Nobuhisa Mizuki, Hideaki Nakajima

**Affiliations:** ^1^ Department of Stem Cell and Immune Regulation, Yokohama City University Graduate School of Medicine, Yokohama, Japan; ^2^ Department of Rheumatology, Endocrinology and Nephrology, Faculty of Medicine and Graduate School of Medicine, Hokkaido University, Sapporo, Japan; ^3^ Department of General Medicine, Kitasato University School of Medicine, Sagamihara, Japan; ^4^ Division of Clinical Nephrology and Rheumatology, Kidney Research Center, Niigata University Graduate School of Medical and Dental Sciences, Niigata, Japan; ^5^ Department of Ophthalmology and Visual Science, Yokohama City University Graduate School of Medicine, Yokohama, Japan; ^6^ Faculty of Health Data Science, Juntendo University, Urayasu, Japan; ^7^ Department of Allergy and Rheumatology, Nippon Medical School Musashi Kosugi Hospital, Kawasaki, Japan

**Keywords:** Behçet disease, disease activity, disease registry, systemic vasculitis, cluster analysis

## Abstract

**Background:**

Little is known about the relationship between the disease activity of Behçet disease (BD) and the incidence of inflammatory major organ events.

**Objectives:**

In this prospective registry study, we investigated the association between the Behçet Disease Current Activity Form (BDCAF) and incidence of inflammatory major organ events, defined as the inflammation of the ocular, central nervous, intestinal, and vascular systems in BD.

**Methods:**

We enrolled participants from Japanese multicenter prospective cohorts. The BDCAF was evaluated annually. BD-related symptoms, including inflammatory major organ events, were monitored. The association between BDCAF and inflammatory major organ events was analyzed by time-to-event analysis. An unsupervised clustering of the participants’ BDCAF, therapeutic agents, and multiple serum cytokines was also performed to examine their association with inflammatory major organ events.

**Results:**

A total of 260 patients were included. The patients had a median BDCAF score of 2 [Interquartile range, 1-3] at the enrolment and remained disease active at 1- and 2-year follow-ups, indicating residual disease activity in BD. Patients with a BDCAF score of 0 had a longer inflammatory major organ event-free survival at 52 weeks than those with a score of 1 or higher (p=2.2 x 10^-4^). Clustering analysis revealed that patients who did not achieve remission despite treatment with tumor necrosis factor inhibitors had high serum inflammatory cytokine levels and incidences of inflammatory major organ events. Among the elevated cytokines, IL-6 was associated with inflammatory major organ events.

**Conclusion:**

This study suggests that treatment strategies targeting overall disease activity and monitoring residual serum IL-6 may help prevent inflammatory major organ events in BD.

## 1 Introduction

Behçet disease (BD) is a severe autoinflammatory condition characterized by a diverse array of clinical manifestations, including recurrent oral and genital ulcers, skin lesions, and joint involvement (1). Among the many symptoms of BD, the most critical ones include lesions affecting the eyes, gastrointestinal systems, blood vessels, and central nervous system, which can result in blindness, organ damage, and even death in severe cases. Patients with these symptoms may have poor prognosis, as indicated by the 2018 European League Against Rheumatism (EULAR) recommendations (2). Predictors of the severity or development of major organ involvement include genetic predisposition, male sex, smoking, elevated levels of serum C-reactive protein, elevated spinal IL-6 levels, elevated fecal calprotectin levels, increased common femoral vein thickness on venous ultrasonography, and fluorescein angiography leakage on retinal angiography (3–10); however, no established method currently exists to accurately predict the occurrence of inflammatory major organ events.

Setting treatment targets is a major obstacle in the management of BD (11). In the case of other autoimmune diseases such as rheumatoid arthritis (RA) and systemic lupus erythematosus, treat-to-target (T2T) strategies using disease activity indicators have been proposed, and achieving therapeutic targets using these strategies has reportedly led to good outcomes (12, 13). Such instances highlight the importance of a disease activity index as a fundamental requirement for T2T implementation. The Outcome Measures in Rheumatology Working Group of BD has also identified disease activity as a crucial domain at the core of mandatory assessments (14).

While standardized criteria for evaluating disease activity in BD have not been established, several disease activity indices have been developed, amongst which the Behçet Disease Current Activity Form (BDCAF) is the most commonly used index (15, 16). Despite its limitations in assigning a low number of scores for major organ involvement and difficulties in quantitatively assessing each symptoms, the BDCAF is a simple assessment that can be easily applied in routine clinical practice. Consequently, the BDCAF is increasingly employed as an endpoint, particularly in clinical studies (17), and is a strong candidate for a disease activity index for T2T.

The 2018 EULAR recommendations state that the desired outcome of BD treatment is the prevention of organ damage. Ideally, the outcome would be organ damage, however, Behcet’s syndrome organ damage index (BODI), an organ damage index for BD recently developed by an international group of European experts and patient representatives, was proved to discriminate damage from disease activity as a confounding factor, and may be inappropriate as an outcome for this study (18, 19). On the other hand, this study also revealed that increased organ damage was linked to a higher recurrence rate of BD symptoms. In other words, inflammatory major organ events can also be regarded as important outcomes of BD and may be appropriate for use as predictive outcomes that can be assessed by the BDCAF.

Herein, we aimed to investigate the relationship between the BDCAF and the incidence of inflammatory major organ events. Although the BDCAF has regulatory approval for use as a secondary outcome in the RELIEF study of apremilast, which included Japanese patients, our investigation started with the BDCAF survey to understand the current situation of BD, as there have been no such cross-sectional studies to date that have involved Japanese patients. Then, we examined the relationship between the incidence of inflammatory major organ events and the BDCAF. Additionally, we performed molecular analyses using cytokine data by unsupervised clustering to gain insights into potential future therapeutic targets.

## 2 Methods

### 2.1 Study design

This study was a prospective analysis conducted using data from the Japanese Multicenter BD Registry. The registry includes both newly onset patients and those in ongoing care, and patient registration was conducted sequentially in routine outpatient clinical practice. The registry was designed to include predetermined observation components, such as age, sex, BD symptoms, treatments, BDCAF scores and face scales, using a common questionnaire to control information bias. These variables were monitored at approximately one-year intervals. At the time of the follow-up survey, we investigated and recorded the recurrence or development of organ involvement, including inflammation in the eyes, central nervous system, blood vessels, and gastrointestinal system, as well as the medical care provided for these events from the previous survey to the current survey. The details of the survey items are listed in **Supplementary Table 1**. Patient data were collected from the hospitals of the following four universities: Yokohama City University (YCU), Niigata University, Hokkaido University, and Kitasato University. DNA samples were collected at the time of enrolment. Additionally, serum samples were collected at the time of enrollment and annually thereafter, concurrently with BDCAF, and were stored. The Ethics Committee of YCU approved this study (A141127010, A170928021). The study protocol was approved by all participating institutions. All samples were collected after obtaining written informed consent according to the Declaration of Helsinki. The study protocol has been registered in the UMIN Trials Registry (UMIN000048089).

#### 2.1.1 Study participants

Data from patients initially enrolled between 1 February 2019 and 28 February 2022 were analyzed. The inclusion criteria in this analysis were as follows: being ≥ 16 years of age, meeting the revised diagnostic criteria established by the Behçet’s Disease Research Committee of Japan in 1987 (20), and having a disease duration of ≥ 6 months.

#### 2.1.2 Disease activity index

The BDCAF 2006 was used as the disease activity index (15). The presence of symptoms caused by BD such as headache, oral ulceration (OU), genital ulcers, erythema, skin pustules, arthralgia, arthritis, nausea/abdominal pain, bloody diarrhea, new active eye symptoms, evidence of new active nervous system involvement, and evidence of new active major vessel involvement was assessed. Each symptom was assigned a score of 1 point if it existed during the 28 days prior to the evaluation and a physician determined that it was related to BD. The BDCAF score ranged from 0 to 12, with higher scores defined as indicating more disease activity compared to lower scores. Additionally, a 7-point face scale, with higher values indicating worse conditions, was collected separately from both physicians and patients to further quantify disease status. The second tracking score was based on the BDCAF score after a lapse of more than seven months since the initial survey.

To assess the variability of BDCAF scores within the same patient, data were collected by recording BDCAF scores during regular outpatient visits (mostly every 1-3 months) from a subset of patients at YCU for up to one year following their third BDCAF assessment. The point at which an inflammatory disease state occurs, enhancing the treatment of BD, was defined as the observation end for BDCAF in the respective patient in the dense BDCAF tracking survey. When two consecutive observation points had different scores, the calculation assumed that half of the observation period was spent on each score. The mean BDCAF score for the follow-up period was calculated by multiplying the BDCAF score by the number of weeks with that score, and then dividing by the total follow-up period. Variation in BDCAF scores was calculated by subtracting the average score during the follow-up period from the score at the time of the third survey, which marked the commencement of intensive BDCAF monitoring.

#### 2.1.3 Outcomes

The primary outcome was set as inflammatory major organ events occurring up to 52 weeks after each BDCAF survey. An inflammatory major organ event was defined as inflammation in the gastrointestinal system, central nervous system, vascular system, or eye that required additional or escalated treatment with colchicine, oral corticosteroids, immunosuppressants, biologic agents, topical steroid therapy, and endoscopic or surgical interventions. The number of weeks from each BDCAF survey to the occurrence of an inflammatory major organ event was recorded.

### 2.2 Time-to-event analysis and classification and regression tree (CART)

To initially investigate the relationship between BDCAF score and inflammatory major organ events, we plotted Kaplan-Meier curves for BDCAF scores of 0, 1, and 2 or higher categories. We employed these categories because BDCAF 2 or higher has been a threshold tentatively considered as active in previous studies (21–23). Subsequently, we established the BDCAF score cut-off for inflammatory major organ events through time-dependent ROC curve analysis (24). Patients were randomly divided into training and test datasets in a 6:4 ratio; the training dataset was used to determine the cut-off value, and the test dataset was used to validate it. Kaplan–Meier curves illustrated inflammatory major organ event probabilities over time, with differences analyzed via the log-rank test. The disparity in restricted mean survival time (RMST) at 52 weeks, accompanied by 95% confidence intervals, was chosen to compare groups, considering potential Cox proportional hazards violations (25). Since there are no previous studies on the annual incidence of inflammatory major organ events, the one-year recurrence rate of ocular lesions treated with tumor necrosis factor inhibitors was used as a reference for sample size calculations for the time-to-event analysis regarding BDCAF and inflammatory major organ events (26). The incidence of ocular lesions in the low risk group was assumed based on the percentage reduction in attacks in the study including patients switched from infliximab to adalimumab (27). Assuming an alpha error of 0.05 (two-sided), a beta error of 0.2, a one-year event rate of 10% for the risk group and 2% for the low risk group, and a ratio of 7:3 for the risk and low risk groups to the total number referring to the proportion of patients who were active state in previous studies using BDCAF as a disease activity measure (21–23), the total number of required events was calculated as 13.7. Based on this, the overall sample size was calculated to be 194 patients: 136 for the risk group and 58 for the low risk group. Assuming a dropout rate of 20%, the required sample size was calculated to be 243 patients. The assumed event numbers in the risk group would likely make multivariate analysis inappropriate; thus, adjustment for confounding was performed by stratification or restriction. We used the Classification and Regression Tree (CART) algorithm (28) to investigate the impact of each symptom comprising the BDCAF on inflammatory major organ events. CART was constructed with survival object as the objective variable. For the complexity parameter, the value with the lowest cross-validation error (xerror) was used to prune the tree.

### 2.3 Genetics and cytokines

We conducted genome-wide SNP genotyping using the Infinium ImmunoArray-24 v2 BeadChip Kit with the standard protocol recommended by Illumina (San Diego, CA, USA). Genotype imputation was conducted using the Michigan Imputation Server (https://imputationserver.sph.umich.edu) with the 1000 Genomes Phase 3 v5 reference panel (http://www.1000genomes.org). The genotyped and imputed SNP data underwent quality control as previously described (29). We extracted only loci that were proven to be BD-sensitive polymorphic loci in the Japanese population from among these SNP data (30–32). Serum cytokine (IL-1β, IFN-α2, IFN-γ, TNF-α, MCP-1, IL-6, IL-8, IL-10, IL-12p70, IL-17A, IL-18, IL-23, and IL-33) levels of patients with BD were measured using LEGENDplex™ Human Inflammation Panel 1 (13-plex, BioLegend, San Diego, CA, USA) during BDCAF score evaluation. One hundred and eleven age-sex-matched healthy human serum samples from the YCU Hospital Biobank were used as controls.

### 2.4 Clustering analysis and differential cytokine expression analysis

Unsupervised clustering was performed on data with continuous variables using the k-means method with Euclidean distance. The k-medoid algorithm with Gower distance, which can handle mixed data, was applied on clustering analysis with mixed data of categorical and continuous variables (33). Consensus clustering was performed to ensure robustness for determing the number of clusters, with 1000 resampling iterations (using 95% of patients/features), and thenumber of k was set from 2 to 10. One patient with proven carcinomatous status was excluded from this analysis. Cytokines with concentrations lower than the limit of detection (LOD) were replaced by the LOD divided by the square root of 2. Batch effect correction was performed using a linear model (34). Since each cytokine concentration had a skewed distribution, it was standardized after log transformation and used as a variable. BDCAF was converted into three categorical variables using an identified classification to predict the inflammatory major organ event up to 52 weeks. TNF inhibitors were treated as a binary categorical variable depending on whether they were used or not. Cytokines with differential expression among clusters were evaluated by false discovery rate, with 0.05 as the threshold. From cytokines showing differential expression between clusters with the highest and lowest incidence of inflammatory major organ events, we selected the cytokine demonstrating the highest predictive accuracy for inflammatory major organ events through time-dependent ROC curves. Using the CART analysis, we examined whether the cytokine and clinical information could serve as predictors for inflammatory major organ events.

### 2.5 Statistical analysis

R version 4.2.2 (http://cran.r-project.org/) was used for the statistical analysis. Continuous variables were presented as mean ± SD or median [IQR] and analyzed using Student’s t-test or Wilcoxon rank-sum test, depending on data normality. Categorical variables were presented as counts and compared using chi-square or Fisher’s exact test. Linear mixed models, incorporating survey time as an independent variable, were employed to examine longitudinal changes in the BDCAF scores. The models also accounted for random effects to accommodate individual variations. Multiple-group comparison was performed with a one-way test or Kruskal-Wallis test for continuous variables and a chi-square test for categorical variables. Values of p <0.05 were considered statistically significant. Various R packages, such as “survivalROC” (35) for time-dependent ROC curve analysis, “survRM2” (36) for RMST analysis, “rpart” (37) for survival CART analysis, “limma” (38) for correcting cytokine batch effect and FDR, “ConsensusClusterPlus” (39) and “cluster” (40) for consensus clustering and clustering respectively, and the “VIM” package (41) for calculating Gower distance, were employed.

## 3 Results

### 3.1 Clinical features of enrolled patients with BD

**Figure 1:**
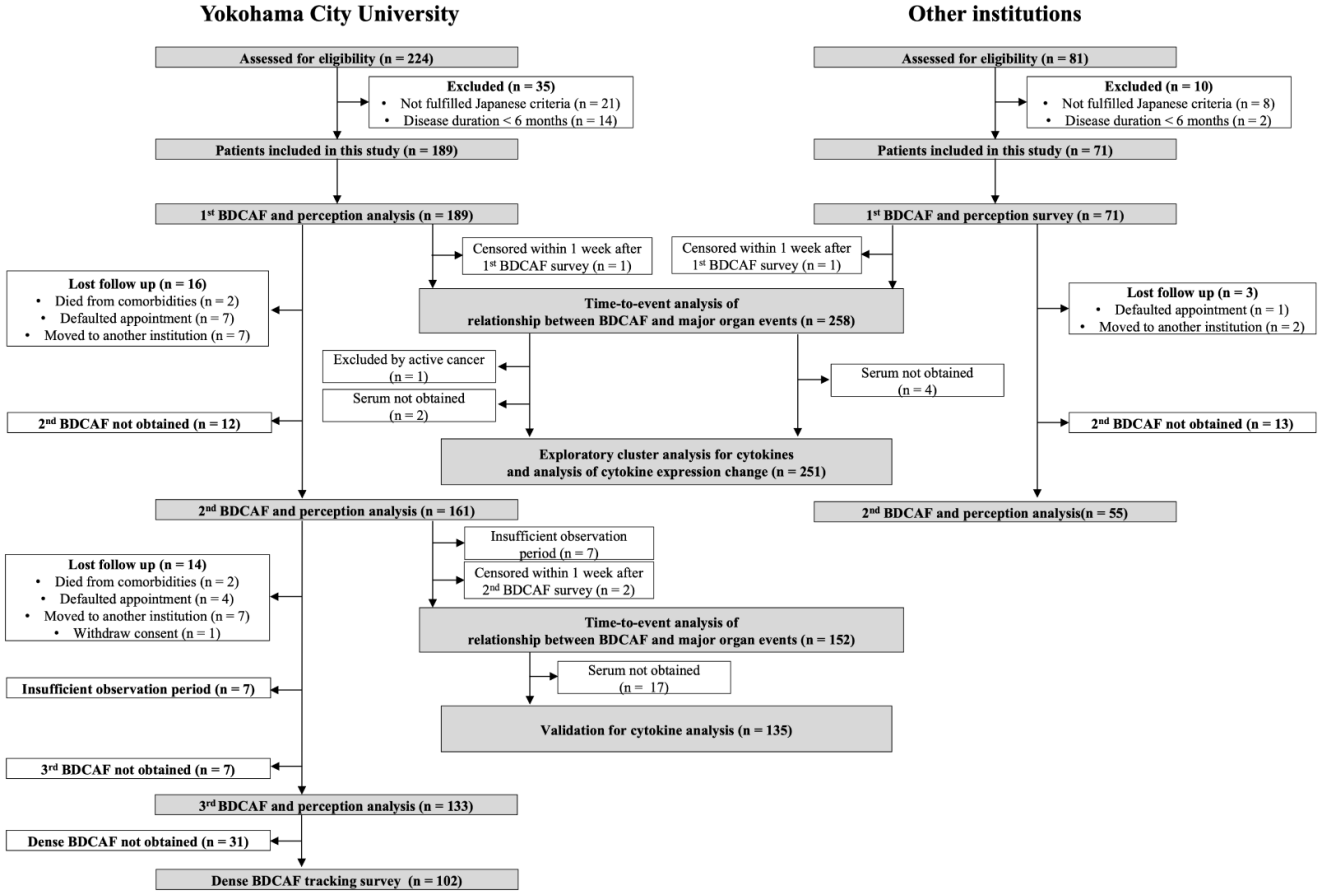
Flow chart of this study. BDCAF, Behçet Disease Current Activity Form.

**Figure 2:**
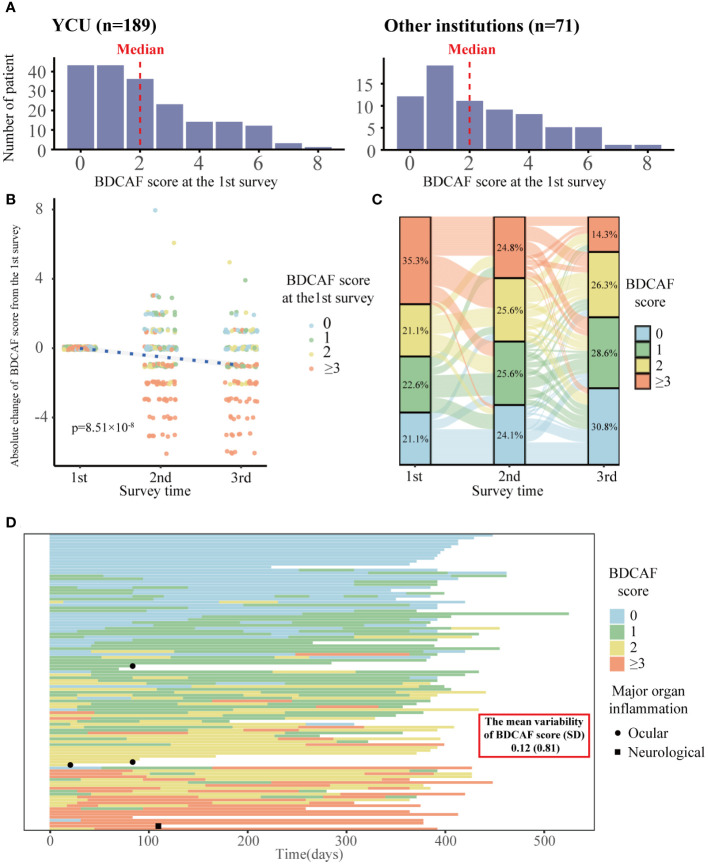
Distribution of BDCAF scores and trends over time in Behçet disaese patients in this study. **(A)** Histogram of BDCAF scores at the 1st survey for YCU of 189 patients and the other institutions of 71 patients. The red dotted line represents the median BDCAF score for each cohort. **(B)** Plot of absolute change in BDCAF scores from the first BDCAF survey, color-coded according to the first BDCAF score. The blue dotted line represents the asymptote; the P-Value shows the p-Value of survey time estimated by a linear mixed model with survey time as the fixed effect and individuals as the random effect. **(C)** Alluvial plot showing the BDCAF scores of the 133 patients who completed up to the third survey. One line represents one patient, color-coded according to BDCAF score, and the percentages represent the percentage of each BDCAF score category at the time of each survey. **(D)** Swimmer plot showing dense BDCAF tracking survey (n=102). One line represents a single patient, color-coded by BDCAF score, and the horizontal axis represents days. BDCAF, Behçet Disease Current Activity Form; SD, Standard deviation.

**Figure 1** displays the flowchart of this study. After screening, 189 and 71 consecutive patients from YCU and the other institutions, respectively, were included in the final analyses. The follow-up rate was 85.2% in the second BDCAF survey, 70.3% in the third survey of YCU, and 77.5% in the second survey of the other institutions. The median duration from the initial BDCAF survey to the second BDCAF survey was 14 months [IQR 9-16].There were no significant differences in age, sex, or disease duration between the two cohorts at enrolment (**Table 1**). The median BDCAF scores at enrolment for both patients from YCU and the other institutions were 2 [IQR, 1–3] and 2 [IQR, 1–4], respectively, indicating that these patients generally had at least two BD symptoms among those listed in the BDCAF (**Figure 2A**). The median patient face scale score in the first survey was 4 [IQR, 2–5] for patients from YCU and 4 [IQR, 3–5] for patients from the other institutions, suggesting that many patients rated themselves as having remaining disease activity (**Supplementary Figure 1**). The most prevalent symptom across the entire sample was OU (**Table 2**). The prevalence of headache, nausea, and diarrhea components differed between YCU and other institutions. Most patients had been receiving medications that had been approved in Japan for the treatment of BD, including colchicine, corticosteroids, immunosuppressants, and biologics. Although BDCAF scores significantly decreased over time, only a limited number of patients reached a BDCAF score of 0 in the second survey in both cohorts (**Figure 2B**, **Supplementary Figure 1**). An alluvial plot of BDCAF scores among the 133 patients who completed the analysis up to the third survey revealed that 62.9% still exhibited BDCAF scores of ≥ 1 in the third survey (**Figure 2C**). A detailed follow-up of the BDCAF for each patient visit revealed that the average range of scores remained stable over a 1-year duration, with a mean score variability of 0.12 [SD, 0.81] (**Figure 2D**). These results indicate that disease activity persisted in the majority of the included Japanese patients with BD, despite the availability of existing medical therapies.

**Table 1:** Characteristics of Behçet disease patients participated in the study.

	All (n=260)	YCU (n=189)	Other institutions (n=71)	P value
Age (mean (SD))	49.21 (14.53)	49.46 (14.89)	48.52 (13.62)	0.643
Sex: male (%)	117 (45)	89 (47.1)	28 (39.4)	0.334
Nationality (%)				0.751
Japanese	255 (98.1)	184 (97.4)	71 (100.0)	
Chinese	2 (0.8)	2 (1.1)	0 (0.0)	
Japanese-French	1 (0.4)	1 (0.5)	0 (0.0)	
Japanese-Pakistan	1 (0.4)	1 (0.5)	0 (0.0)	
Japanese-American	1 (0.4)	1 (0.5)	0 (0.0)	
Disease duration, year (mean (SD))	13.43 (11.32)	13.56 (11.33)	13.09 (11.37)	0.767
Organ involvement present from onset to registration				
Oral ulceration (%)	258 (99.2)	188 (99.5)	70 (98.6)	1
Genital ulcer (%)	176 (67.7)	130 (68.8)	46 (64.8)	0.642
Skin lesion (%)	240 (92.3)	176 (93.1)	64 (90.1)	0.587
Erythema nodosum like lesion (%)	159 (61.2)	114 (60.3)	45 (63.4)	0.758
Pseudo folliculitis (%)	176 (67.7)	136 (72)	40 (56.3)	0.024
Joint involvement (%)	173 (66.5)	127 (67.2)	46 (64.8)	0.827
Ocular involvement (%)	148 (56.9)	109 (57.7)	39 (54.9)	0.797
Neurological involvement (%)	30 (11.5)	23 (12.2)	7 (9.9)	0.763
Vascular lesion (%)	24 (9.2)	18 (9.5)	6 (8.5)	0.979
Gastrointestinal lesion (%)	61 (23.5)	40 (21.2)	21 (29.6)	0.207
International diagnostic criteria				
Fulfilled ISG criteria (%)	234 (90.0)	172 (91.0)	62 (87.3)	0.516
Fulfilled ICBD criteria (%)	256 (98.5)	187 (98.9)	69 (97.2)	0.645

YCU, Yokohama City University; SD, standard deviation; ISG, International Study Group; ITR-ICBD, International Team for the Revision of the International Criteria for Behçet’s Disease, IQR, Interquartile range; BDCAF, Behçet Disease Current Activity Form; TNF, Tumor necrosis factor; 5-ASA, 5-aminosalicylic acid.

**Table 2:** Disease activity of Behçet disaase patients participated in the study.

	All (n=260)	YCU (n=189)	Other institutions (n=71)	P value
BDCAF (median [IQR])	2.0 [1.0, 3.0]	2.0 [1.0, 3.0]	2.0 [1.0, 4.0]	0.413
Patient face scale (median [IQR])	4.0 [2.0, 5.0]	4.0 [2.0, 5.0]	4.0 [3.0, 5.0]	0.052
Physician face scale (median [IQR])	3.0 [2.0, 4.0]	3.0 [2.0, 4.0]	3.0 [2.0, 4.0]	0.121
BDCAF component				
Headache (%)	87 (33.5)	49 (25.9)	38 (53.5)	<0.001
Oral ulceration (%)	136 (52.3)	106 (56.1)	30 (42.3)	0.064
Genital ulcer (%)	24 (9.2)	19 (10.1)	5 (7.0)	0.612
Erythema (%)	46 (17.7)	35 (18.5)	11 (15.5)	0.699
Skin pustule (%)	61 (23.5)	50 (26.5)	11 (15.5)	0.090
Joints-Arthralgia (%)	106 (40.8)	76 (40.2)	30 (42.3)	0.875
Joints-Arthritis (%)	20 (7.7)	14 (7.4)	6 (8.5)	0.984
Nausea/vomiting/abdominal pain (%)	55 (21.2)	33 (17.5)	22 (31.0)	0.027
Diarrhea + altered/frank blood per rectum (%)	19 (7.3)	8 (4.2)	11 (15.5)	0.005
New active eye symptom (%)	21 (8.1)	17 (9.0)	4 (5.6)	0.528
Evidence of new active nervous system involvement (%)	6 (2.3)	4 (2.1)	2 (2.8)	1.000
Evidence of new active major vessel inflammation (%)	0 (0.0)	0 (0.0)	0 (0.0)	NA
Medication use at the survey				
Colchicine (%)	166 (63.8)	124 (65.6)	42 (59.2)	0.412
TNF inhibitors (%)	87 (33.5)	58 (30.7)	29 (40.8)	0.162
Corticosteroid (%)	62 (23.8)	40 (.2)	22 (31.0)	0.136
Immunosuppressant (%)	76 (29.2)	54 (28.6)	22 (31.0)	0.819
Methotrexate (%)	27 (10.4)	15 (7.9)	12 (16.9)	0.06
5-ASA (%)	38 (14.6)	26 (13.8)	12 (16.9)	0.658
Azathioprine (%)	15 (5.8)	13 (6.9)	2 (2.8)	0.341
Calcineurin inhibitors (%)	8 (3.1)	7 (3.7)	1 (1.4)	0.581
Apremilast (%)	1 (0.4)	0 (0.0)	1 (1.4)	0.61
No systemic treatment (%)	33 (12.7)	27 (14.3)	6 (8.5)	0.294

YCU, Yokohama City University; SD, standard deviation; IQR, Interquartile range; BDCAF, Behçet Disease Current Activity Form; TNF, Tumor necrosis factor; 5-ASA, 5-aminosalicylic acid.

### 3.2 Association between BD disease activity and incidence of inflammatory major organ events

**Figure 3:**
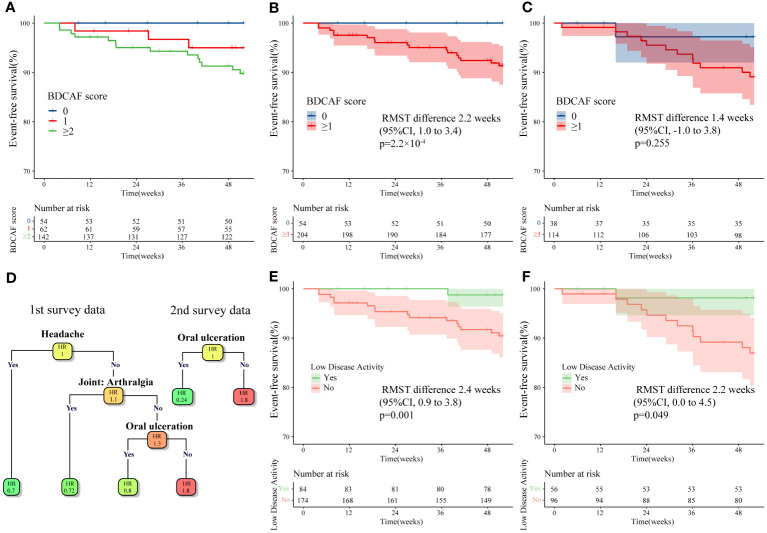
Kaplan–Meier curve and survival decision tree analysis for inflammatory major organ events in Behçet disease patients in this study. **(A)** Kaplan–Meier curves for the first BDCAF survey data (n=258) stratified by BDCAF score 0, 1, ≥2. **(B)** Kaplan–Meier curves for the first BDCAF survey data (n=258) stratified by BDCAF score 0 or ≥1. **(C)** Kaplan–Meier curves for the second BDCAF survey data (n=152) in the same patients as **(B)** stratified by BDCAF score 0 or ≥1. **(D)** Survival decision trees were constructed using Classification and regression tree (CART) algorithm with each symptom as a variable for the first BDCAF survey data (n=258) and the second BDCAF survey data (n=152). The number in each node represents the relative hazard ratio (HR), with darker orange of node indicating higher HR and the darker green of node indicating lower HR. **(E)** Kaplan–Meier curve of the first BDCAF survey data (n=258), which divides patients into two groups; “low disease activity” or not. Low disease activity is defined as patients with BDCAF score 0 or only OU. **(F)** The Kaplan-Meier curve constructed for the definition of ‘low disease activity’ applied to the second BDCAF survey data (n=152). BDCAF, Behçet disease current activity form; CI, confidence intervals; RMST, Restricted mean survival time, EFS, Event-free survival, HR, Hazard ratio.

Our data revealed remaining disease activity in a substantial number of Japanese patients with BD, although the clinical significance of these residual scores is unclear as BD has no clear treatment goal. To clarify the significance of the remaining BDCAF, we investigated the relationship between BDCAF scores in the first survey and event-free survival (EFS) for the incidence of inflammatory major organ events, an important treatment target for BD. For time-to-event analysis, data up to 52 weeks after the first BDCAF survey were collected from all facilities, while data up to 52 weeks after the second BDCAF survey were obtained from YCU. We initially performed a time-to-event analysis of the outcomes stratified into three categories of BDCAF scores: 0, 1, and ≥ 2. We found that patients with a BDCAF score ≥ 2 had a higher incidence of inflammatory major organ events (**Figure 3A**). Subsequently, we sought to identify the cut-off value of the BDCAF score for the occurrence of inflammatory major organ events, based on a time-dependent ROC curve with the training dataset. The optimal cut-off value for the BDCAF score was 1 (**Supplementary Figure 2**), and the patients were divided into two groups (BDCAF 0 and BDCAF ≥1).

In the time-to-event analysis, we observed a significant difference in EFS in both the training and test datasets (**Supplementary Figure 2**) and found that the BDCAF 0 group had significantly longer EFS than the BDCAF ≥1 groups in the combined dataset (RMST difference 2.2 weeks; 95%CI 1.0–3.4; p=2.2 x 10^-4^, **Figure 3B**). A similar trend was observed in the data of up to 52 weeks from the second BDCAF survey (**Figure 3C**). No independent variables associated with inflammatory major organ events, other than the BDCAF, were identified based on the univariate analysis of age, sex, and treatment (**Table 3**).

**Table 3:** Time-to-event analysis or time until the occurrence of inflammatory major organ events.

	No. of events/ total no, (%)	RMST, weeks	Difference in RMST (95%CI)	p-value
Total events	17/258 (6.6)	–	–	–
Ocular	11/258 (4.3)	–	–	–
Gastrointestinal	4/258 (1.6)	–	–	–
Neurological	1/258 (0.4)	–	–	–
Vascular	1/258 (0.4)	–	–	–
Variables included in the model				
BDCAF				
BDCAF 0	0/54 (0.0)	52.0	2.3 (1.0 to 3.4)	2.2×10^-4^
BDCAF ≥ 1	17/204 (8.3)	49.8		
Age				
Age < 50	7/123 (5.7)	50.0	-0.6 (-2.4 to 1.3)	0.564
Age ≥ 50	10/135 (7.4)	50.5		
Sex				
Male	10/117 (8.5)	50.1	-0.3 (-2.2 to 1.6)	0.762
Female	7/141 (5.0)	50.4		
Colchicine				
Yes	9/165 (5.5)	50.9	1.8 (-0.5 to 4.1)	0.117
No	8/93 (8.6)	49.1		
TNF inhibitor				
Yes	7/87 (8.0)	49.5	-1.1 (-3.4 to 1.1)	0.325
No	10/171 (5.8)	50.6		
Corticosteroid				
Yes	6/62 (9.7)	49.3	-1.3 (-3.9 to 1.4)	0.345
No	11/196 (5.6)	50.5		
Immunosuppressant				
Yes	5/76 (6.6)	50.6	0.6 (-1.2 to 2.4)	0.525
No	12/182 (6.6)	50.1		

RMST, Restricted mean survival time; BDCAF, Behçet Disease Current Activity Form; TNF, Tumor necrosis factor.

Additionally, we compared the clinical features between the BDCAF 0 and BDCAF ≥1 groups. In the BDCAF 0 group, a higher prevalence of men (70.4% vs. 38.7%, p=6.338×10^-5^) and a higher rate of TNF inhibitor use (50.0% vs. 29.4%, p=0.007 were noted (**Supplementary Table 2**). It is possible that this is because males are known to have more severe forms of the disease, and that the BDCAF 0 group includes more patients who were severely ill at the onset of the disease but are now at BDCAF 0 due to the appropriate use of TNF inhibitors. We performed stratified analyses to address the potential confounding effects of these variables; however, significant disparities in EFS between the BDCAF 0 and BDCAF ≥1 groups persisted, even within each subgroup (**Supplementary Figure 3A, B**).

Finally, since patients without pre-existing major organ involvement are presumed to have a lower risk of experiencing inflammatory major organ events, we limited the time-to-event analysis of the poor prognosis subtype to patients with ocular, intestinal, neurological, or vascular disease. In the current study, the sole occurrence of an inflammatory major organ event among patients without pre-existing major organ diseases involved an individual who developed acute neuro-inflammation after the second BDCAF survey. Moreover, considering the possibility of a stable group of patients with long-term disease, we restricted our analysis to patients with below-average disease duration.(**Supplementary Figure 3D**). In addition, we limited the sub-analysis to those who met the ISG and ICBD criteria(**Supplementary Figure 3E, F**). The results showed that in all subgroup analyses, the BDCAF 0 group had significantly longer EFS than the BDCAF ≥1 group. Collectively, our findings suggest a relationship between disease activity and inflammatory major organ events in BD.

### 3.3 Relationship between specific BDCAF items and prognosis

Although BDCAF 0 was defined as ‘remission’, patients with mild symptoms can also be classified as having ‘non-remission’, and thereby potentially confound the analysis. Hence, in patients with only one residual symptom, 12 components of the BDCAF were assessed using a survival decision tree to determine whether any specific BDCAF items were permissible. The survival decision tree analysis of the first survey data revealed that ‘headache’, ‘joint: arthralgia’, and ‘OU’ were acceptable symptoms; analysis using the second survey data identified only ‘OU’ as an acceptable symptom (**Figure 3D**). Based on this, residual OU only was considered acceptable and the BDCAF score of 0 or residual OU only was tentatively defined as ‘Low disease activity (LDA)’. Following this classification, the patients were divided into two groups (LDA or not LDA), and EFS was compared using Kaplan–Meier curves. The group with LDA showed a significantly longer EFS (**Figure 3E**) compared to not LDA group. The same criteria were then applied to data from the second survey, and the same results were obtained (**Figure 3F**).

### 3.4 Molecular mechanisms associated with BD disease activity

**Figure 4:**
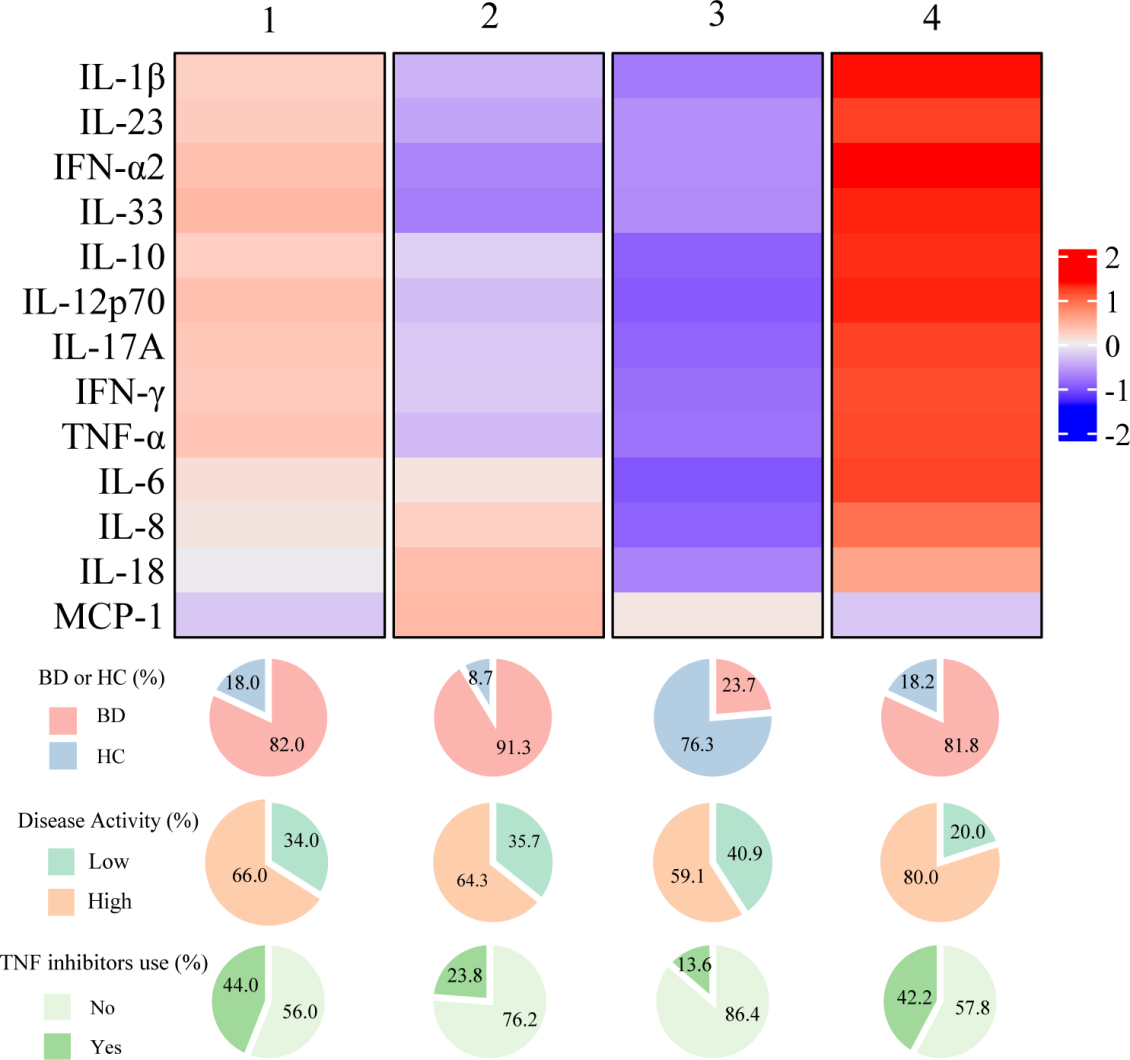
Summarized heatmap depicting serum cytokine concentration using combined data from Behçet disease patients and healthy subjects. The pie charts below the heatmap depict the percentage of BD patients and healthy controls within each cluster (top), as well as the distribution of disease activity and TNF inhibitor usage among BD patients in each cluster (middle and bottom). BD, Behçet disease; HC, Healthy controls; TNF, Tumor necrosis factor.

To obtain molecular insights into patients at risk of inflammatory major organ events of BD, an unsupervised cluster analysis was performed on the cytokine profile in the combined data of healthy controls and patients with BD. Four clusters were obtained (**Supplementary Figure 4**), and while a small number of patients were found to have cytokine profiles similar to those of healthy controls, the other three clusters were mostly composed of patients with BD, indicating that the cytokine profiles of patients with BD are classifiable (**Figure 4**). Notably, the group with higher overall cytokine levels showed a higher percentage of TNF inhibitor usage, compared to others, despite no significant differences in the percentage of patients who had pre-existing major organ involvement (**Supplementary Table 3**).

**Figure 5:**
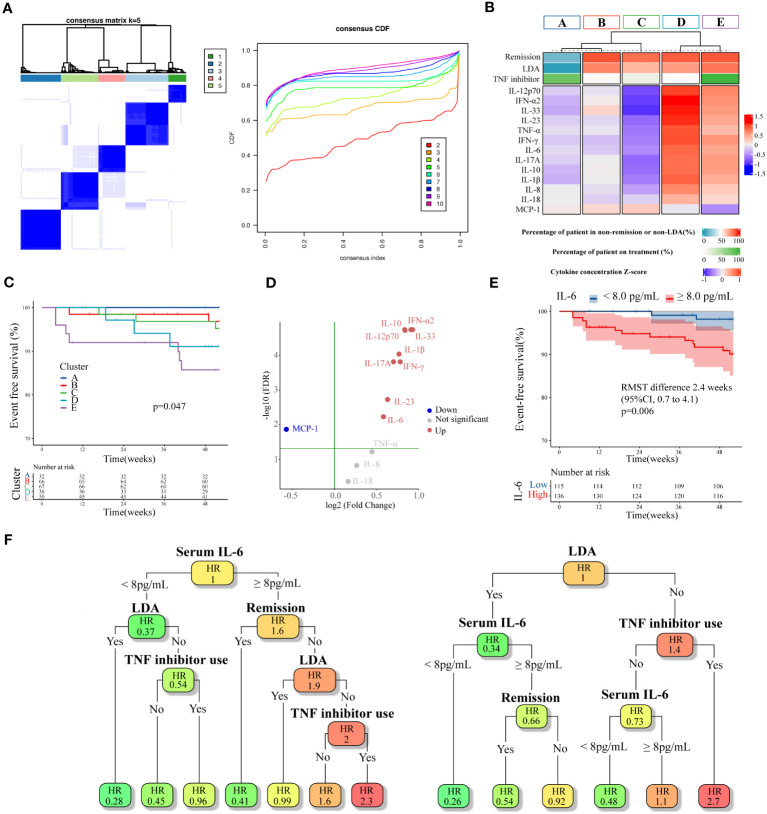
Cluster analysis of cytokines combined with BDCAF and therapeutic agents, and exploration of expression variation cytokines. **(A)** Heatmap of consensus matrices of k=5 (left). The same patients are symmetrically placed in rows and columns. The right is a Cumulative Distribution Function (CDF) plot, where the x-axis is the consensus index, and the y-axis is the cumulative percentage of patients. **(B)** Summarized heatmap depicting serum cytokine concentrations and percentage of non-remission (BDCAF ≥ 1) and percentage of TNF inhibitor use for each cluster. Cytokines are expressed as a Z-score. **(C)** Kaplan–Meier curves for each cluster. **(D)** A Volcano plot illustrating the difference in cytokine expression between Cluster A and E. The vertical axis represents log10FDR, with the green line indicating an FDR of 0.05. **(E)** Kaplan–Meier curves for the first BDCAF survey data (n=251) stratified by serum IL-6 concentration. **(F)** pruned survival decision trees with the minimum cross-validation error constructed for the first survey data (n=251, left), and the second survey data (n=135, right). Disease activity, TNF inhibitors use, and serum IL-6 concentration was used as variable. In each node, color indicates relative hazard ratio (HR), with darker orange signifying higher and darker green suggesting lower. BDCAF, Behçet disease current activity form; TNF, Tumor necrosis factor; IL, Interleukin; IFN, Interferon; MCP, Macrophage chemotactic protein.

Based on these findings, we hypothesized that there is a group at high risk for inflammatory major organ events that exhibited residual disease activity and upregulated inflammatory cytokines even with the use of TNF inhibitors. Subsequently, we performed an exploratory clustering analysis to attempt a detailed classification based on the cytokine profiles, the BDCAF scores, and TNF inhibitors. The consensus clustering results showed that k=5 was stable (**Figure 5A**). By implementing clustering using the k-medoid method, patients were divided into five clusters, of which Cluster E had the highest incidence of inflammatory major organ events (**Figures 5B, C**). In Cluster E (characterized by a predominantly male population with a high prevalence of past ocular lesions), the number of patients who had a BDCAF score of 0 was the lowest (10.0%) as compared to that in other clusters, despite all patients receiving TNF inhibitors (**Supplementary Table 4**). Furthermore, this cluster exhibited higher inflammatory cytokine concentrations as compared to the mean concentration of the overall dataset. In contrast, in Cluster A (characterized by the lowest occurrence of inflammatory major organ events) 84.4% of the patients had a BDCAF score of 0, and the levels of serum cytokine concentrations were lower as compared to those in other clusters (**Figure 5A**, **Supplementary Table 4**). None of the evaluated BD-associated SNPs were significantly enriched in Cluster E (**Supplementary Table 5**).

Finally, as concentrations of several inflammatory cytokines were elevated in Cluster E, we attempted to identify which of these cytokines were most closely associated with inflammatory major organ events, with the aim of facilitating clinical interpretation. The analysis of cytokine expression variability in Cluster E and Cluster A revealed that IL-33, IL-10, IFN-α2, IL-12p70, IL-1β, IFN-γ, IL-17A, IL-23, and IL-6 were upregulated in Cluster E compared to Cluster A, while MCP-1 was downregulated (**Figure 5D**). These cytokines also exhibited variable expression when comparing Cluster E with Cluster B and Cluster C. As a result, these cytokines were considered potential candidates for assessing their correlation with inflammatory major organ events through survival ROC analysis. Among them, IL-6 displayed the highest predictive accuracy (AUC=0.633). with a cut-off value of 2.13 log pg/mL, translated to 8 pg/mL for practical clinical interpretation. A survival CART constructed using disease activity, TNF inhibitor usage, and serum IL-6 concentration revealed that the node with the greatest relative hazard ratio (HR) corresponded to the group with residual disease activity despite the use of TNF inhibitors and a serum IL-6 concentration above the cut-off. The group featuring elevated serum IL-6 levels beyond the cut-off, ongoing disease activity, and absence of TNF inhibitors, all of which seemed to correspond to Cluster D, exhibited the second-highest relative HR. When this algorithm and variables were applied to the second survey data, the node displaying high disease activity even with TNF inhibitor usage yielded the highest relative HR. Furthermore, when disease activity was high without TNF inhibitors, higher serum IL-6 concentrations above the cut-off were associated with higher relative HRs, which was consistent with the primary analysis. These data suggest that patients at risk of inflammatory major organ events may show upregulation of the autoinflammatory pathways, despite treatment with TNF inhibitors, making serum IL-6 levels a potential future biomarker.

## 4 Discussion

The current study revealed that low BDCAF scores were associated with longer EFS in BD. Although the BDCAF was developed to measure ‘current’ BD disease activity, it may serve as a predictor of future inflammatory major organ events. Our findings suggest that a treatment strategy focusing on reducing the BDCAF score to near-zero could be promising. Moreover, our machine learning-based analysis suggests that the presence of residual symptoms being limited to OU at the time of observation may not be associated with the future occurrence of inflammatory major organ events.

This prospective study also revealed that the cytokine profile is associated with future clinical outcomes. Cytokine analysis identified Cluster E, which was characterized by high levels of multiple cytokines, to be associated with inflammatory major organ events. This cluster was considered a clinically difficult-to-treat population, with residual symptoms despite the use of TNF inhibitors. Among the upregulated cytokines, IL-6 was found to be associated with inflammatory major organ events. In consideration of the immunopathology of BD, while BD has aspects of acquired immune system disease, including MHC-class1, it also has aspects of innate immune system disease, as TLR4 and NOD2, which are involved in innate immunity, have been identified as disease susceptibility genes. IL-6 is a pleiotropic cytokine that acts on both innate and acquired immunity (42, 43). The association between elevated serum IL-6 levels and disease activity in BD patients is controversial, though, an association between elevated serum levels of IL-6 and high disease activity has been reported in BD patients, particularly those with ocular lesions (44, 45). Moreover, increased IL-6 level in cerebrospinal fluid is known to be related to neurological prognosis in neuro-BD patients (46, 47). IL-6 also plays a crucial role in Th17 differentiation. Concerning the elevation of Th17 in the blood of BD, an increase in IL-17-producing CD^4^T cells has been observed in BD patients with active uveitis (48). This aligns with the findings of the present study, where increased IL-6 concentrations were implicated in the development of inflammatory major organ events. While the results of the current study do not conclusively determine whether IL-6 is a cause or a consequence, they suggest that it may play an important role in the immunopathology of major organ inflammation. Currently, TNF inhibitors serve as the primary treatment for refractory major organ inflammation in BD (2). However, considering the high incidence of inflammatory major organ events in patients with elevated serum IL-6 levels, even when using TNF inhibitors, therapy targeting IL-6 may be considered as a potential next step. As support for this, the efficacy of anti-IL-6 receptor antibodies have been recently reported to be effective against BD not only in TNF inhibitor naive patients, but even in TNF inhibitor-experienced patients (49, 50).

As the T2T approach for BD has not yet been established, and treatment decisions are made at the discretion of the attending physician, this prospective study did not employ a T2T approach or interventions during treatment to reduce BDCAF scores. Indeed, it is necessary to create remission criteria that also consider therapeutic agents. For example, in systemic lupus erythematosus, the criterion for remission includes adequate corticosteroid reduction. Therefore, future interventional studies should aim to establish the efficacy of a T2T approach in reducing the occurrence of unfavorable outcomes by specifically targeting reduction in BDCAF scores compared to conventional care. In addition, a detailed assessment of individual symptoms, for example, the number of OUs per month or a comprehensive measure of skin lesions similar to the psoriasis area and severity index (51), could further clarify the extent of symptom acceptability. This would define a more accurate low disease activity status, and evidence-based and stratified treatment approaches can be achieved in BD that are similar to the intensified therapy used to achieve remission in RA (52).

Whether the BDCAF is the most suitable treatment target for BD remains uncertain, as there are multiple disease activity measures available, such as the Behçet’s syndrome activity score (BSAS) (53). In the present study, we found that the BDCAF is a clinically practical activity index for BD with good reproducibility and minimal measurement errors across facilities, which can be applied as a first-line tool for assessing disease activity in BD. Our data are intended to serve as a reference for future advancements in T2T research. Meanwhile, the BDCAF is based on a 0,1-rating based on the presence or absence of symptoms and thus lacks a detailed evaluation of symptoms, whereas the BSAS can evaluate the number of OUs and skin lesions. In this study, we did not find any differences in clinical symptoms between Clusters B and C. However, it remains possible that differences could have been detected if the severity of the individual lesions had been considered. To test this possibility, it is necessary to simultaneously measure these indicators with the BDCAF in the future.

This study had several limitations. First, owing to the small number of cases and short observation period, we were unequipped to detect an association between the BDCAF and inflammatory events in each organs and life prognosis. Multivariate analysis could not be performed due to the small number of events. The sample size calculation is partly based on experience due to the lack of previous studies. Furthermore, caution should be exercised when extrapolating the results to patients without pre-existing major organ involvement, as inflammatory major organ events were concentrated in the group with pre-existing major organ involvement. Although consecutive patients were enrolled in this registry, a selection bias cannot be ruled out because patients with milder symptoms might not have been included due to the nature of the study conducted at the university hospitals. In addition, precise evaluation of the disease activity of major organ involvement (such as intestinal fiberscope findings) was not possible. The study design also overlooked several important elements, including quality-of-life indicators, which were not assessed. Because the observation point only occurred once a year in this study, it may be necessary to conduct more frequent and detailed observations in future studies to identify the factors contributing to the suppression of inflammatory major organ events, such as duration of remission. It is also known that environmental factors, such as diet and smoking, are involved, and a more detailed analysis of the extent of exposure to these factors may be necessary. Studies conducted in countries other than Japan may have yielded different results. Furthermore, as the serum substances we focused on were primarily inflammatory cytokines, the molecular mechanisms involved in disease activity such as fibrosis and other inflammatory markers have not yet been fully investigated.

In summary, the present study shows that clinical findings and cytokine profiles are associated with future clinical outcomes.

## Data availability statement

The data presented in the study are deposited in the European Variation Archive (EVA) repository (https://www.ebi.ac.uk/eva/), accession number PRJEB74512.

## Ethics statement

The studies involving humans were approved by the Ethics Committee of Yokohama City University. The studies were conducted in accordance with the local legislation and institutional requirements. Written informed consent for participation in this study was provided by the participants’ legal guardians/next of kin.

## Author contributions

LH: Conceptualization, Data curation, Formal Analysis, Investigation, Visualization, Writing – original draft, Writing – review & editing, Methodology, Validation. YK: Conceptualization, Funding acquisition, Investigation, Project administration, Writing – original draft, Writing – review & editing, Methodology, Supervision, Validation. YS: Data curation, Investigation, Writing – review & editing, Conceptualization, Methodology, Validation. YI: Writing – review & editing, Conceptualization, Data curation, Investigation, Methodology. RY: Writing – review & editing, Investigation, Supervision. YF: Investigation, Supervision, Writing – review & editing. TA: Investigation, Supervision, Writing – review & editing. TT: Investigation, Supervision, Writing – review & editing. DK: Investigation, Supervision, Writing – review & editing. AM: Formal Analysis, Investigation, Writing – review & editing. MaT: Conceptualization, Supervision, Writing – review & editing. KS: Formal Analysis, Supervision, Writing – review & editing. MiT: Conceptualization, Supervision, Writing – review & editing. NM: Supervision, Writing – review & editing. HN: Supervision, Writing – review & editing.
