# Supplementary material for: Association of high disease activity and serum IL-6 levels with the incidence of inflammatory major organ events in Behçet disease: a prospective registry study
Source: Front Immunol. 2024 Apr 15;15:1354969. doi: 10.3389/fimmu.2024.1354969 (PMC11057327; doi:10.3389/fimmu.2024.1354969)
Supplement: Supplementary file 1 [file DataSheet_1.docx]

Supplementary Material

Association of high disease activity and serum IL-6 levels with the incidence of inflammatory major organ events in Behçet disease: a prospective registry study

**Lisa Hirahara MD ^1^, Yohei Kirino MD, PhD ^1*^, Yutaro Soejima MD, PhD ^1^, Yuki Iizuka MD ^1^, Ryusuke Yoshimi MD, PhD ^1^, Yuichiro Fujieda MD, PhD ^2^, Tatsuya Atsumi MD, PhD ^2^, Toshihiro Tono MD, PhD ^3^, Daisuke Kobayashi MD, PhD ^4^, Akira Meguro PhD ^5^, Masaki Takeuchi MD, PhD ^5^, Kentaro Sakamaki PhD ^6^, Mitsuhiro Takeno MD, PhD ^7^, Nobuhisa Mizuki MD, PhD ^5^, Hideaki Nakajima MD, PhD ^1^**

*** Correspondence:** Yohei Kirino, MD, PhD: [kirino@yokohama-cu.ac.jp](mailto:kirino@yokohama-cu.ac.jp)

**Supplementary Tables**

**Supplementary Table 1: List of survey items**

| Items at the initial survey | |
| --- | --- |
| Baseline characteristics | **Objective examination findings** |
| ・Birth date | ・Pathergy |
| ・Sex | ・HLA-B51 |
| ・Familial history of Behçet’s disease | ・HLA-A26 |
| ・First diagnostic date | ・Concentration of cerebrospinal fluid (CSF) IL-6 |
| ・First visit of the institution | ・Brain MRI findings |
| ・Last visit date | ・CSF cell counts |
| ・Nationality | **Medications used up to the time of the survey** |
| ・Living condition | *Start and end dates |
| ・Activities of Daily Living (ADL) | ・Colchicine |
| ・Social history | ・Corticosteroids |
| - Smoking, Alcohol | - Max dose of prednisolone or equivalent |
| ・Living condition | - Pulse of methylprednisolone |
| Clinical course until the time of survey | ・Cyclosporine |
| *Onset of lesions and Activity of past one year | ・Methotrexate |
| ・Oral ulceration | ・Azathioprine |
| ・Skin lesion | ・5-ASA |
| ・Ocular lesion | ・Other medications |
| ・Genital ulcer | ・Infliximab |
| ・Arthritis | ・Adalimumab |
| ・Spondylitis/ Enthesitis | ・Other biologics |
| ・Epididymitis | ・Local corticosteroids |
| ・Gastrointestinal involvement | ・Anti-coagulants |
| - Surgery | **Medical history and comorbidities** |
| ・Vascular involvement | ・Number of hospitalizations and days until the time of the survey |
| - Surgery | **Disease activity index** |
| ・Neurological involvement | ・BDCAF |
|  | ・Face scale: patient & physician |

| Items at the follow-up survey | |
| --- | --- |
| Outcome | **Change of medication from past survey to survey** |
| ・Continue or Discontinue of survey (reason) | *Change dates and reason |
| ・Hospitalization from previous survey to survey | ・Colchicine |
| ・Change in ADLs from previous survey to survey | ・Corticosteroids |
| ・Blindness from previous survey to survey | - Max dose of prednisolone or equivalent |
| Relapse of major organ from previous survey to survey | ・Apremilast |
| *Date of relapse/ treatment and outcome | ・Cyclosporine |
| ・Ocular lesion | ・Methotrexate |
| ・Gastrointestinal involvement | ・Azathioprine |
| ・Vascular involvement | ・5-ASA |
| ・Neurological involvement | ・Others |
| New lesions from previous survey to survey | ・Infliximab |
| *Date of onset | ・Adalimumab |
| ・Oral ulceration | ・Other biologics |
| ・Skin lesion | **Disease activity index** |
| ・Ocular lesion | ・BDCAF |
| ・Genital ulcer | ・Face scale: patient & physician |
| ・Arthritis |  |
| ・Spondylitis/ Enthesitis |  |
| ・Epididymitis |  |
| ・Gastrointestinal involvement |  |
| - Surgery |  |
| ・Vascular involvement |  |
| - Surgery |  |
| ・Neurological involvement |  |

TNF, Tumor necrosis factor; 5-ASA, 5-aminosalicylic acid; PSL, Prednisolone; BDCAF: Behçet Disease Current Activity Form, Hospitalization is defined as related to Behcet disease, including cases not necessarily linked to inflammatory major organ events.

**Supplementary table 2: Comparison of baseline characteristics between BDCAF score 0 and ≥1 group**

|  | **BDCAF 0** | **BDCAF ≥1** | **P value** |
| --- | --- | --- | --- |
| n | 54 | 204 |  |
| Age (mean (SD)) | 49.36 (13.49) | 49.41 (14.68) | 0.981 |
| Sex: male (%) | 38 (70.4) | 79 (38.7) | 6.338×10^-5^ |
| Past organ involvement | | | |
| Ocular involvement (%) | 34 (63.0) | 114 (55.9) | 0.435 |
| Neurological involvement (%) | 10 (18.5) | 20 (9.8) | 0.124 |
| Gastrointestinal lesion (%) | 11 (20.4) | 50 (24.5) | 0.648 |
| Vascular lesion (%) | 3 (5.6) | 21 (10.3) | 0.422 |
| None of above (%) | 8 (14.8) | 42 (20.6) | 0.447 |
| Medication used at the survey | | | |
| TNF inhibitors (%) | 27 (50.0) | 60 (29.4) | 0.007 |
| Colchicine (%) | 28 (51.9) | 137 (67.2) | 0.054 |
| Corticosteroid (%) | 15 (27.8) | 47 (23) | 0.585 |
| Immunosuppressant (%) | 14 (25.9) | 62 (30.4) | 0.637 |

BDCAF, Behçet Disease Current Activity Form; SD, Standard deviation; TNF, Tumor necrosis factor

| Cluster | 1 | 2 | 3 | 4 | P-Value |
| --- | --- | --- | --- | --- | --- |
| n | 100 | 84 | 22 | 45 |  |
| Age (mean (SD)) | 47.35 (14.64) | 52.90 (14.47) | 51.31 (12.61) | 45.99 (14.12) | 0.02 |
| Sex: male (%) | 45 (45.0) | 39 (46.4) | 10 (45.5) | 21 (46.7) | 0.997 |
| Remission; BDCAF=0 (%) | 22 (22.0) | 19 (22.6) | 6 (27.3) | 5 (11.1) | 0.335 |
| BDCAF (median [IQR]) | 2.00 [1.00, 3.00] | 2.00 [1.00, 3.00] | 1.00 [0.25, 2.75] | 2.00 [1.00, 4.00] | 0.349 |
| Patient's face scale (median [IQR]) | 4.00 [2.00, 5.00] | 4.00 [3.00, 5.00] | 4.00 [3.00, 4.75] | 4.00 [3.00, 5.00] | 0.947 |
| Physician's face scale (median [IQR]) | 3.00 [2.00, 4.00] | 3.00 [2.00, 4.00] | 2.00 [2.00, 4.00] | 3.00 [2.00, 4.00] | 0.158 |
| Headache (%) | 30 (30.0) | 23 (27.4) | 9 (40.9) | 20 (44.4) | 0.179 |
| Oral ulceration (%) | 57 (57.0) | 45 (53.6) | 8 (36.4) | 24 (53.3) | 0.378 |
| Genital ulcer (%) | 11 (11.0) | 6 (7.1) | 1 (4.5) | 6 (13.3) | 0.534 |
| Erythema (%) | 17 (17.0) | 18 (21.4) | 3 (13.6) | 8 (17.8) | 0.804 |
| Skin pustule (%) | 21 (21.0) | 20 (23.8) | 3 (13.6) | 16 (35.6) | 0.165 |
| Joints-Arthralgia (%) | 41 (41.0) | 28 (33.3) | 9 (40.9) | 24 (53.3) | 0.182 |
| Joints-Arthritis (%) | 8 (8.0) | 7 (8.3) | 2 (9.1) | 3 (6.7) | 0.984 |
| Nausea/vomiting/abdominal pain (%) | 19 (19.0) | 18 (21.4) | 4 (18.2) | 12 (26.7) | 0.748 |
| Diarrhea + altered/frank blood per rectum (%) | 8 (8.0) | 7 (8.3) | 2 (9.1) | 1 (2.2) | 0.562 |
| New active eye symptom (%) | 9 (9.0) | 5 (6.0) | 1 (4.5) | 5 (11.1) | 0.667 |
| New active nervous system involvement (%) | 0 (0.0) | 4 (4.8) | 0 (0.0) | 2 (4.4) | 0.12 |
| TNF inhibitors (%) | 44 (44.0) | 20 (23.8) | 3 (13.6) | 19 (42.2) | 0.003 |
| Colchicine (%) | 59 (59.0) | 55 (65.5) | 15 (68.2) | 33 (73.3) | 0.388 |
| Corticosteroid (%) | 21 (21.0) | 23 (27.4) | 1 (4.5) | 12 (26.7) | 0.125 |
| Immunosuppressant (%) | 28 (28.0) | 25 (29.8) | 5 (22.7) | 14 (31.1) | 0.901 |
| Inflammatory major organ events (%) | 9 (9.0) | 2 (2.4) | 1 (4.5) | 3 (6.7) | 0.298 |
| **Past organ involvement** | | | | | |
| Ocular involvement (%) | 62 (62.0) | 43 (51.2) | 15 (68.2) | 27 (60.0) | 0.355 |
| Neurological involvement (%) | 13 (13.0) | 11 (13.1) | 1 (4.5) | 4 (8.9) | 0.621 |
| Gastrointestinal lesion (%) | 24 (24.0) | 20 (23.8) | 4 (18.2) | 10 (22.2) | 0.943 |
| Vascular lesion (%) | 7 (7.0) | 10 (11.9) | 3 (13.6) | 3 (6.7) | 0.535 |

**Supplementary Table 3: Comparison of characteristics for cytokine clusters in Behçet disease patients and healthy controls**

BDCAF, Behçet Disease Current Activity Form, SD, Standard deviation, TNF, Tumor necrosis factor

**Supplementary Table 4: Characteristics for each cluster**

|  | A | B | C | D | E | P value |
| --- | --- | --- | --- | --- | --- | --- |
| n | 32 | 66 | 67 | 36 | 50 |  |
| Age (mean (SD)) | 46.05(11.29) | 49.68(15.00) | 55.14(14.54) | 45.57(13.83) | 45.78(14.12) | 0.001 |
| Sex: male (%) | 26(81.2) | 21(31.8) | 24(35.8) | 12(33.3) | 32(64.0) | <0.001 |
| Remission; BDCAF=0 (%) | 27(84.4) | 6(9.1) | 10(14.9) | 4(11.1) | 5(10.0) | <0.001 |
| BDCAF (median [IQR]) | 0.00[0.00,0.00] | 2.00[1.00,4.00] | 2.00[1.00,3.00] | 2.00[1.00,4.00] | 2.00[1.00,4.00] | <0.001 |
| Patient's face scale (median [IQR]) | 2.00[1.00,3.00] | 4.00[3.00,5.00] | 4.00[3.00,5.00] | 4.00[2.50,4.00] | 4.00[3.00,5.00] | <0.001 |
| Physician's face scale (median [IQR]) | 1.50[1.00,3.00] | 3.00[2.00,4.00] | 3.00[2.00,4.00] | 2.50[2.00,4.00] | 4.00[3.00,5.00] | <0.001 |
| Headache (%) | 1(3.1) | 22(33.3) | 22(32.8) | 14(38.9) | 23(46.0) | 0.002 |
| Oral ulceration (%) | 5(15.6) | 41(62.1) | 39(58.2) | 21(58.3) | 28(56.0) | <0.001 |
| Genital ulcer (%) | 0(0.0) | 10(15.2) | 4(6.0) | 5(13.9) | 5(10.0) | 0.109 |
| Erythema (%) | 0(0.0) | 15(22.7) | 12(17.9) | 7(19.4) | 12(24.0) | 0.058 |
| Skin pustule (%) | 0(0.0) | 19(28.8) | 14(20.9) | 13(36.1) | 14(28.0) | 0.005 |
| Joints-Arthralgia (%) | 0(0.0) | 28(42.4) | 27(40.3) | 19(52.8) | 28(56.0) | <0.001 |
| Joints-Arthritis (%) | 0(0.0) | 9(13.6) | 6(9.0) | 2(5.6) | 3(6.0) | 0.178 |
| Nausea/vomiting/abdominal pain (%) | 0(0.0) | 15(22.7) | 13(19.4) | 9(25.0) | 16(32.0) | 0.013 |
| Diarrhea + altered/frank blood per rectum (%) | 0(0.0) | 7(10.6) | 5(7.5) | 2(5.6) | 4(8.0) | 0.428 |
| New active eye symptom (%) | 0(0.0) | 3(4.5) | 5(7.5) | 3(8.3) | 9(18.0) | 0.03 |
| Evidence of new active nervous system involvement (%) | 0(0.0) | 1(1.5) | 2(3.0) | 0(0.0) | 3(6.0) | 0.311 |
| TNF inhibitors (%) | 26(81.2) | 2(3.0) | 8(11.9) | 0(0.0) | 50(100.0) | <0.001 |
| Colchicine (%) | 12(37.5) | 46(69.7) | 48(71.6) | 26(72.2) | 30(60.0) | 0.008 |
| Corticosteroid (%) | 8(25.0) | 16(24.2) | 13(19.4) | 8(22.2) | 12(24.0) | 0.957 |
| Immunosuppressant (%) | 7(21.9) | 22(33.3) | 18(26.9) | 8(22.2) | 17(34.0) | 0.565 |
| Past Organ involvement |  |  |  |  |  |  |
| Ocular involvement (%) | 24(75.0) | 27(40.9) | 38(56.7) | 19(52.8) | 39(78.0) | <0.001 |
| Neurological involvement (%) | 7(21.9) | 6(9.1) | 7(10.4) | 2(5.6) | 7(14.0) | 0.252 |
| Gastrointestinal lesion (%) | 5(15.6) | 23(34.8) | 12(17.9) | 6(16.7) | 12(24.0) | 0.091 |
| Vascular lesion (%) | 2(6.2) | 7(10.6) | 7(10.4) | 3(8.3) | 4(8.0) | 0.947 |

SD, Standard deviation, BDCAF, Behçet Disease Current Activity Form, IQR, Interquartile range; TNF, Tumour necrosis factor

**Supplementary Table 5: Comparison of disease susceptibility gene risk SNP prevalence by cluster (n=209)**

|  | A | B | C | D | E | P value |
| --- | --- | --- | --- | --- | --- | --- |
| n | 29 | 53 | 57 | 29 | 41 |  |
| ***IL12RB2, IL23R (%)*** | | | | | | **0.8** |
| C/C | 1(3.4) | 4(7.5) | 5(8.8) | 4(13.8) | 1(2.4) |  |
| C/T | 12(41.4) | 18(34.0) | 20(35.1) | 10(34.5) | 17(41.5) |  |
| T/T | 16(55.2) | 31(58.5) | 32(56.1) | 15(51.7) | 23(56.1) |  |
| ***IL10 (%)*** | | | | | | **0.2** |
| C/C | 2(6.9) | 2(3.8) | 8(14.0) | 1(3.4) | 0(0.0) |  |
| C/T | 12(41.4) | 22(41.5) | 18(31.6) | 10(34.5) | 19(46.3) |  |
| T/T | 15(51.7) | 29(54.7) | 31(54.4) | 18(62.1) | 22(53.7) |  |
| ***IL1A, IL1B (%)*** | | | | | | ***0.228*** |
| T/T | 1(3.4) | 4(7.5) | 4(7.0) | 3(10.3) | 2(4.9) |  |
| G/T | 7(24.1) | 22(41.5) | 28(49.1) | 9(31.0) | 22(53.7) |  |
| G/G | 21(72.4) | 27(50.9) | 25(43.9) | 17(58.6) | 17(41.5) |  |
| ***TFCP2L1* (%)** | | | | | | **0.36** |
| C/C | 25(86.2) | 46(86.8) | 53(93.0) | 25(86.2) | 34(82.9) |  |
| A/C | 4(13.8) | 7(13.2) | 4(7.0) | 3(10.3) | 7(17.1) |  |
| A/A | 0(0.0) | 0(0.0) | 0(0.0) | 1(3.4) | 0(0.0) |  |
| ***STAT4* (%)** | | | | | | 0.858 |
| C/C | 4(13.8) | 8(15.1) | 14(24.6) | 5(17.2) | 6(14.6) |  |
| A/C | 18(62.1) | 33(62.3) | 31(54.4) | 15(51.7) | 23(56.1) |  |
| A/A | 7(24.1) | 12(22.6) | 12(21.1) | 9(31.0) | 12(29.3) |  |
| ***CCR1-CCR3*(%)** | | | | | | **0.109** |
| C/C | 24(82.8) | 42(79.2) | 51(89.5) | 18(62.1) | 30(73.2) |  |
| C/T | 5(17.2) | 10(18.9) | 6(10.5) | 11(37.9) | 11(26.8) |  |
| T/T | 0(0.0) | 1(1.9) | 0(0.0) | 0(0.0) | 0(0.0) |  |
| ***ERAP1*(%)** | | | | | | **0.619** |
| C/T | 2(6.9) | 5(9.4) | 3(5.3) | 1(3.4) | 5(12.2) |  |
| ***HLA-A26*(%)** | | | | | | **0.236** |
| None | 16(55.2) | 39(73.6) | 43(75.4) | 22(75.9) | 24(58.5) |  |
| Hetero | 13(44.8) | 13(24.5) | 13(22.8) | 6(20.7) | 17(41.5) |  |
| Homo | 0(0.0) | 1(1.9) | 1(1.8) | 1(3.4) | 0(0.0) |  |
| ***HLA-B51*(%)** | | | | | | **0.572** |
| None | 15(51.7) | 37(69.8) | 33(57.9) | 21(72.4) | 24(58.5) |  |
| Hetero | 11(37.9) | 14(26.4) | 20(35.1) | 8(27.6) | 15(36.6) |  |
| Homo | 3(10.3) | 2(3.8) | 4(7.0) | 0(0.0) | 2(4.9) |  |
| ***IFNGR1*(%)** | | | | | | **0.432** |
| T/T | 25(86.2) | 45(84.9) | 52(91.2) | 28(96.6) | 39(95.1) |  |
| C/T | 4(13.8) | 8(15.1) | 4(7.0) | 1(3.4) | 2(4.9) |  |
| C/C | 0(0.0) | 0(0.0) | 1(1.8) | 0(0.0) | 0(0.0) |  |
| ***RIPK2*(%)** | | | | | | **0.449** |
| C/C | 29(100.0) | 52(98.1) | 53(93.0) | 27(93.1) | 41(100.0) |  |
| C/T | 0(0.0) | 1(1.9) | 3(5.3) | 2(6.9) | 0(0.0) |  |
| T/T | 0(0.0) | 0(0.0) | 1(1.8) | 0(0.0) | 0(0.0) |  |
| **Intergenic *LNCAROD/DKK1*(%)** | | | | | | 0.897 |
| A/A | 17(58.6) | 34(64.2) | 33(57.9) | 14(48.3) | 25(61.0) |  |
| A/T | 10(34.5) | 14(26.4) | 19(33.3) | 13(44.8) | 14(34.1) |  |
| T/T | 2(6.9) | 5(9.4) | 5(8.8) | 2(6.9) | 2(4.9) |  |
| ***ADO-EGR2*(%)** | | | | | | 0.364 |
| G/G | 5(17.2) | 8(15.1) | 8(14.0) | 8(27.6) | 5(12.2) |  |
| A/G | 10(34.5) | 27(50.9) | 34(59.6) | 12(41.4) | 21(51.2) |  |
| A/A | 14(48.3) | 18(34.0) | 15(26.3) | 9(31.0) | 15(36.6) |  |
| **Intergenic *JRKL/CNTN5*(%)** | | | | | | 0.14 |
| C/C | 5(17.2) | 17(32.1) | 25(43.9) | 10(34.5) | 16(39.0) |  |
| C/T | 20(69.0) | 33(62.3) | 22(38.6) | 16(55.2) | 20(48.8) |  |
| T/T | 4(13.8) | 3(5.7) | 10(17.5) | 3(10.3) | 5(12.2) |  |
| ***KLRC4*(%)** | | | | | | 0.349 |
| C/C | 20(69.0) | 21(39.6) | 25(43.9) | 12(41.4) | 18(43.9) |  |
| C/T | 8(27.6) | 25(47.2) | 27(47.4) | 13(44.8) | 20(48.8) |  |
| T/T | 1(3.4) | 7(13.2) | 5(8.8) | 4(13.8) | 3(7.3) |  |
| ***LACC1*(%)** | | | | | | 0.799 |
| C/C | 13(44.8) | 33(62.3) | 29(50.9) | 16(55.2) | 23(56.1) |  |
| C/G | 12(41.4) | 17(32.1) | 24(42.1) | 10(34.5) | 16(39.0) |  |
| G/G | 4(13.8) | 3(5.7) | 4(7.0) | 3(10.3) | 2(4.9) |  |
| ***IRF8*(%)** | | | | | | 0.646 |
| C/C | 0(0.0) | 1(1.9) | 0(0.0) | 0(0.0) | 1(2.4) |  |
| C/G | 6(20.7) | 11(20.8) | 8(14.0) | 3(10.3) | 4(9.8) |  |
| G/G | 23(79.3) | 41(77.4) | 49(86.0) | 26(89.7) | 36(87.8) |  |
| **Intergenic *CEBPB/PTPN1*(%)** | | | | | | 0.015 |
| T/T | 1(3.4) | 9(17.0) | 11(19.3) | 0(0.0) | 2(4.9) |  |
| C/T | 17(8.6) | 22(41.5) | 15(26.3) | 15(51.7) | 18(43.9) |  |
| T/T | 11(37.9) | 22(41.5) | 31(54.4) | 14(48.3) | 21(51.2) |  |

SNP, Single nucleotide polymorphism

**Supplementary Figure 1: Distribution of patient face scale at the first survey and BDCAF score at the second survey**


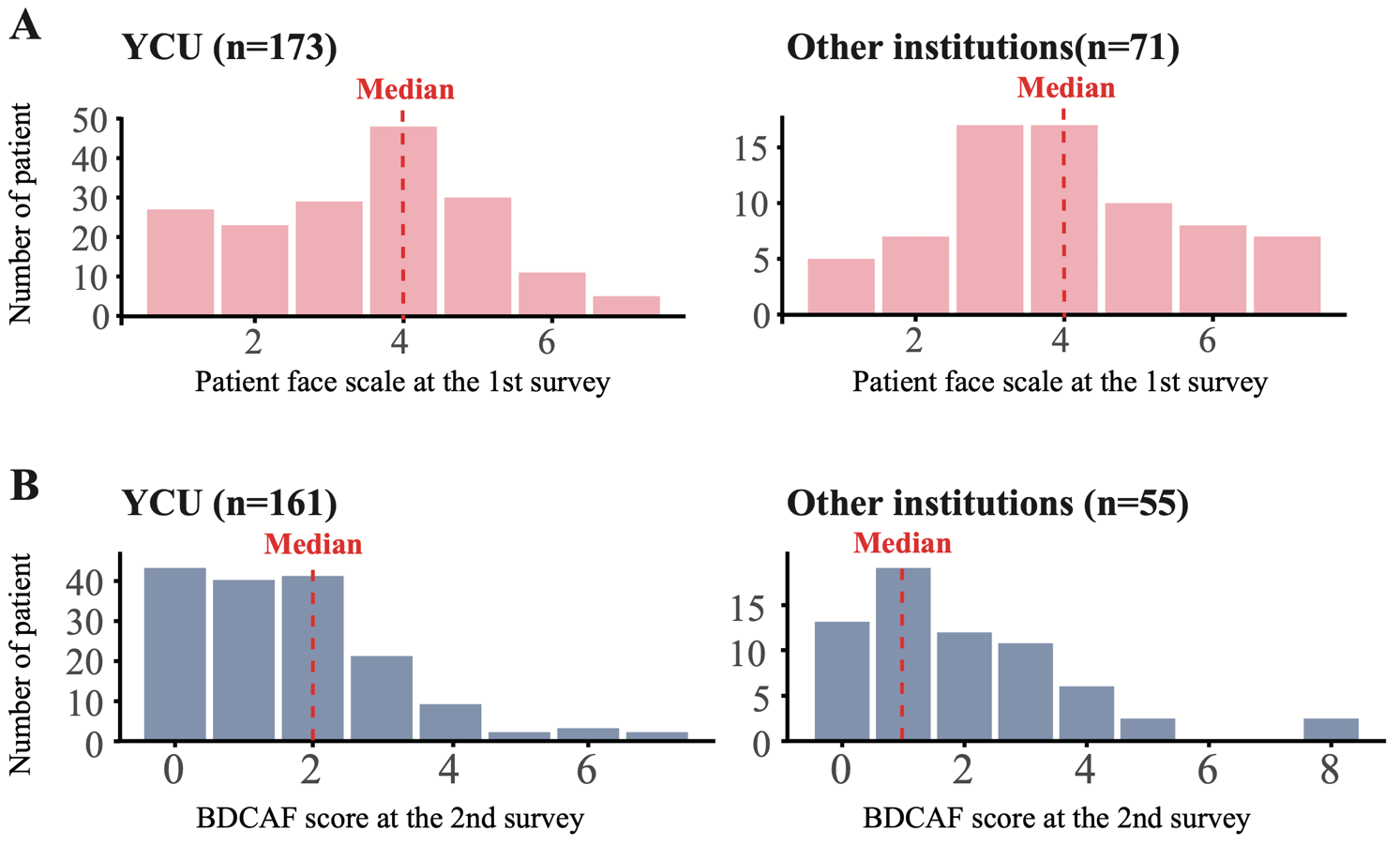


A, Histogram of patient face scale at the first survey for YCU of 173 patients and other institutions of 71 patients. The red dotted line represents the median of patient face scale for each cohort.

**Supplementary Figure 2: Time-dependent ROC analysis and Kaplan-Meier curve in the model randomly divided into training and test set**

**
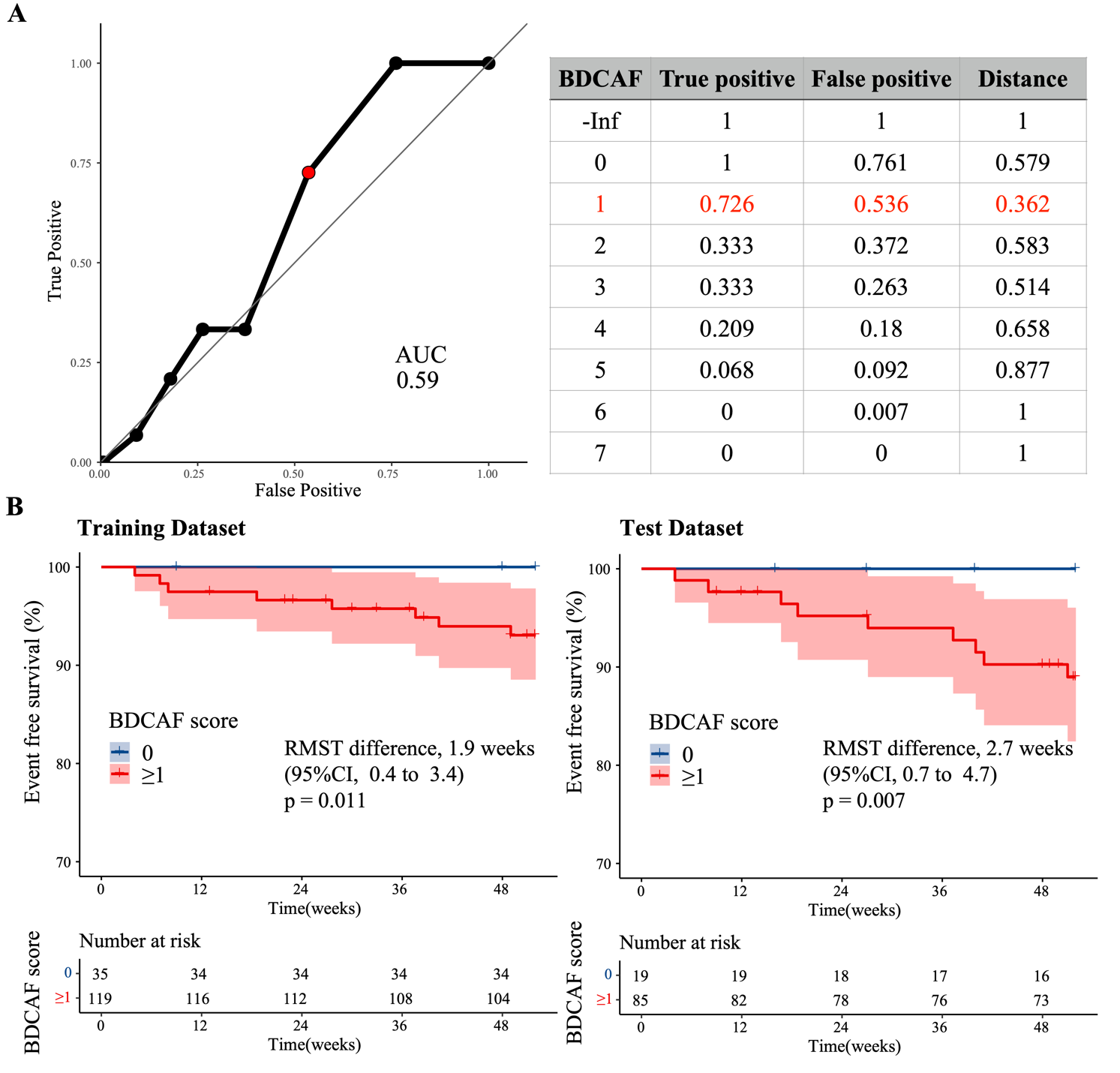
**

A, the ROC curve and the table of the true positive rate, false positive rate, and distance to the top left corner of the ROC curve for each cutoff value using the training dataset (n=154). The cutoff value with the lowest distance was found to be BDCAF score 1, and the point with BDCAF score 1 is shown in red point in the ROC curve. B, Kaplan-Meier curve of time until inflammatory major organ events for the training data set (n=154) and the test data set (n=104). In both data sets, BDCAF 0 group showed significantly longer event-free survival than the BDCAF ≥ 1 group. AUC, Area under curve; BDCAF, Behçet Disease Current Activity Form; TP, True positive; FP, False positive; RMST, Restricted mean survival time; CI, Confidence interval

**Supplementary Figure 3: Kaplan-Meier curve of time until inflammatory major organ events for sub-analysis**

**
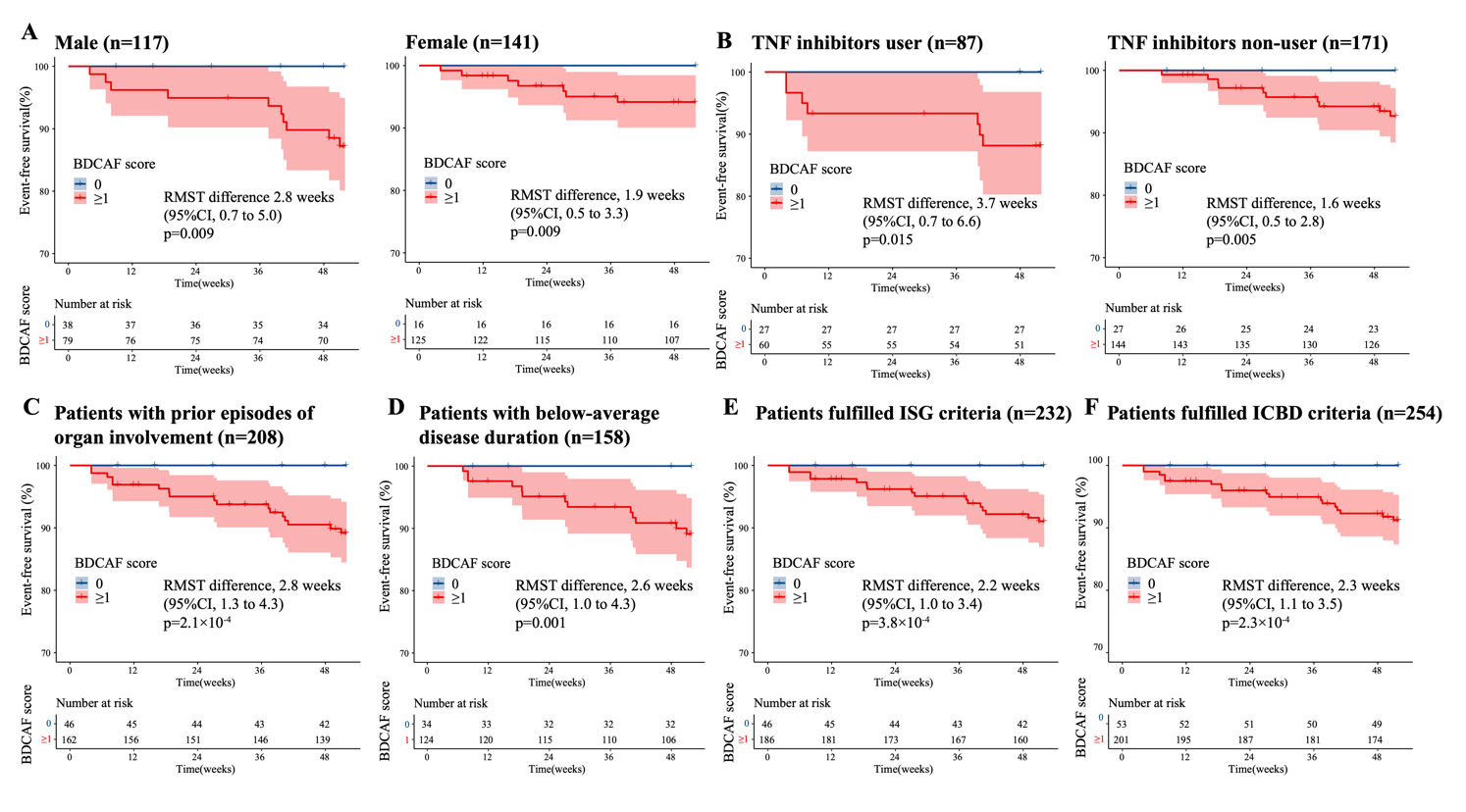
**

A, Stratified analysis by gender: 117 males and 141 females. In both strata, BDCAF 0 group showed significantly longer event-free survival than the BDCAF 1 group.

B, Stratified analysis by TNF inhibitor use: 87 TNF inhibitor users and 171 non-users. In both strata, BDCAF 0 group showed significantly longer event-free survival than the BDCAF 1 group. C, Analysis focused on patients with previous major organ involvement (n=208). D, Analysis limited to patients with below-average disease duration (n=158). E, Analysis limited to patients meeting ISG criteria (n=232). F, Analysis limited to patients meeting ICBD criteria (n=254). BDCAF, Behçet Disease Current Activity Form; RMST, Restricted mean survival time; CI, Confidence interval; ISG, International Study Group; ICBD, International Criteria for Behçet’s Disease.

**Supplementary Figure 4: Heatmap of consensus matrices and cumulative distribution function of cytokine clustering in Behçet disease and healthy controls**

**
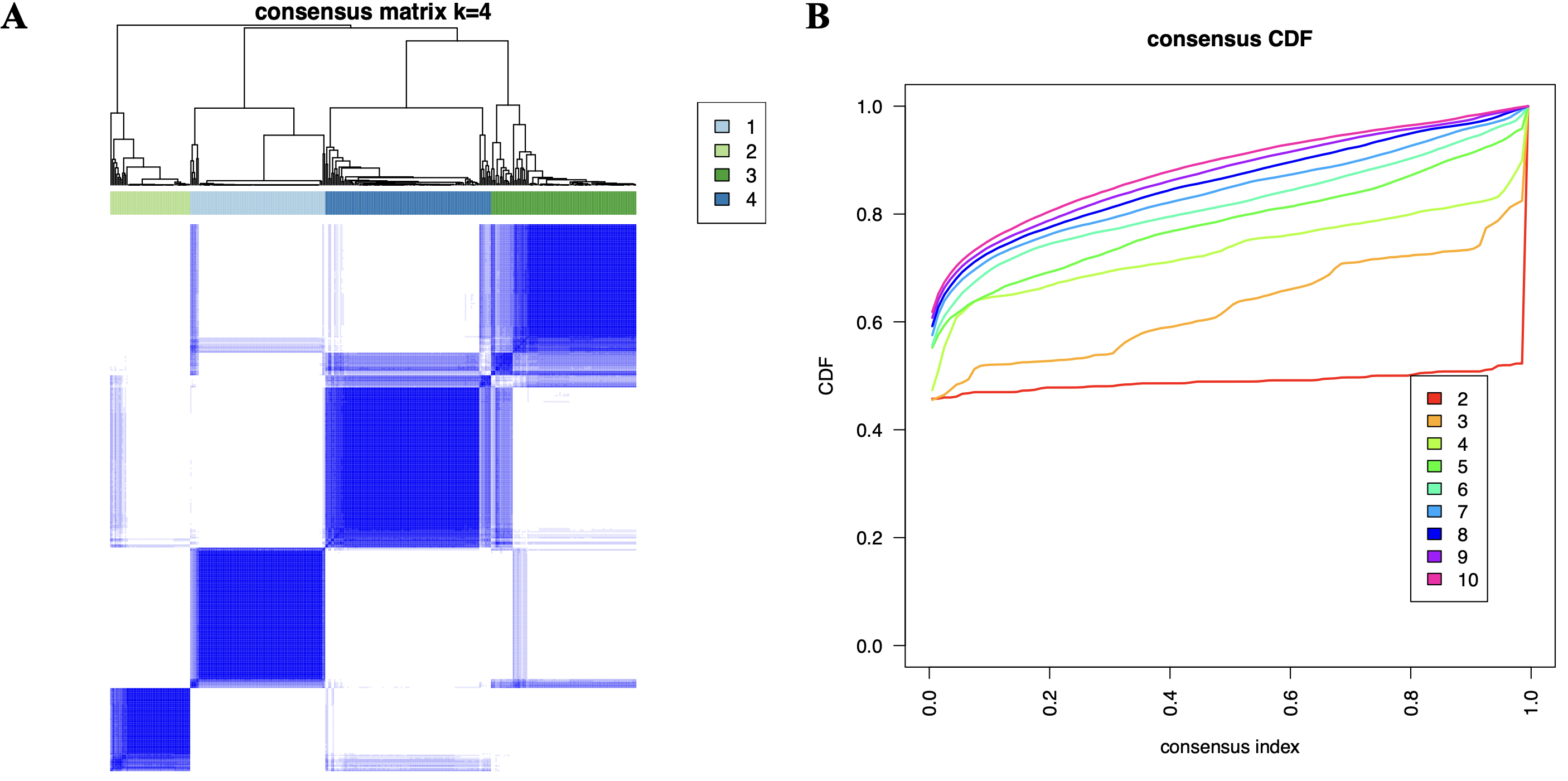
**

A, Heatmap of consensus matrices of k=4 is shown. The same patients are symmetrically placed in rows and columns. B, the above panel is a Cumulative Distribution Function (CDF) plot, where the x-axis is the consensus index and the y-axis is the cumulative percentage of patients, where the consensus index is a measure of how well patients are clustered into the same cluster during the sampling run. The clustering is more stable when there are many 0s and 1s and no intermediate values, since 0 means never clustered together and 1 means always clustered together. From this plot, the k-optima are estimated to be 4.
